# Treatment of Early Trilogy Valve Aortic Insufficiency With Valve‐in‐Valve Transcatheter Aortic Valve Replacement

**DOI:** 10.1155/cric/6773549

**Published:** 2026-08-24

**Authors:** Johan van Nispen, Michael Massoomi, Eric Jeng

**Affiliations:** ^1^ Department of Surgery, Division of Cardiac Surgery, University of Florida, Gainesville, Florida, USA, ufl.edu; ^2^ Department of Medicine, Division of Cardiology, University of Florida, Gainesville, Florida, USA, ufl.edu

**Keywords:** TAV in TAV, Trilogy valve, ViV TAVR

## Abstract

Aortic insufficiency (AI) is the third most common left valvular heart disease. Transcatheter aortic valve replacement (TAVR) is becoming popular in the treatment of AI. The Trilogy Valve (JenaValve Technology, Irvine, California, United States) is in clinical trials. We report here a case of valve‐in‐valve TAVR with a Sapien S3 Ultra Resilia valve (Edwards Lifesciences Corporation, Irvine, California, United States) for recurrent AI after Trilogy valve placement.

## 1. Introduction

Aortic insufficiency (AI) is the third most common left valvular heart disease and often is present in patients with multiple comorbidities. As such, the transcatheter aortic valve replacement (TAVR) has become increasingly popular for the treatment of AI [1] in off‐label uses for patients unsuitable for surgical aortic valve replacement (SAVR). More recently, the Trilogy valve (JenaValve Technology, Irvine, California, United States) is in clinical trials and has been approved in Europe. This is a self‐expanding, supra‐annular porcine valve that has shown promising results in the ALIGN‐AR trial [2]. With increase in TAVR for both aortic stenosis (AS) and AI, the incidence of prosthetic failure has risen, prompting a rise in “Valve‐in‐Valve” (ViV) TAVR as an alternative to surgical explant and replacement [3]. Surgeons and interventionalists must understand how to treat a degenerated Trilogy valve. We report a case of ViV TAVR in the Trilogy valve for recurrent AI.

## 2. Case

The patient is a 70‐year‐old male with a history of coronary artery disease with multiple percutaneous interventions, a prior coronary artery bypass graft, and AI who had a 25‐mm Trilogy valve implanted 1 year. He presented with dyspnea on exertion and intermittent left sided chest pain for 1 week. Transthoracic echocardiogram (TTE) was performed with concern for moderate to severe bioprosthetic AI. Transesophageal echocardiogram (TEE) revealed severe transvalvular regurgitation with holodiastolic flow reversal in the descending aorta, pressure half time of 270 ms, and additional moderate paravalvular leak (Figure 1A). The right and noncoronary leaflets appeared to move properly; however, the left appeared to be smaller and did not coapt well leading to the transvalvular regurgitation. Operative reports were not available, so whether the valve was dilated is unknown. The valve appeared to have asymmetric expansion though, leading to early failure. No stenosis was observed. Computed tomography imaging was performed demonstrating annular size of 27 mm (Figure 1B) and left main coronary artery (LMCA) height of 21.6 mm and right coronary height of 24.5 mm. Cardiac catheterization demonstrated patent left internal mammary‐left anterior descending and vein graft‐diagonal bypasses as well as open circumflex stents. These findings were consistent with early valve failure. After multidisciplinary valve conference, the patient was offered ViV TAVR with a 29‐mm Edwards Sapien S3 Ultra Resilia valve (Edwards Lifesciences Corporation, Irvine, California, United States).

Figure 1(A) TEE demonstration of severe AI of the Trilogy valve with paravalvular regurgitation. (B) Preoperative computed tomography imaging for ViV TAVR planning. Annulus size measures 27 mm.

**Figure:**
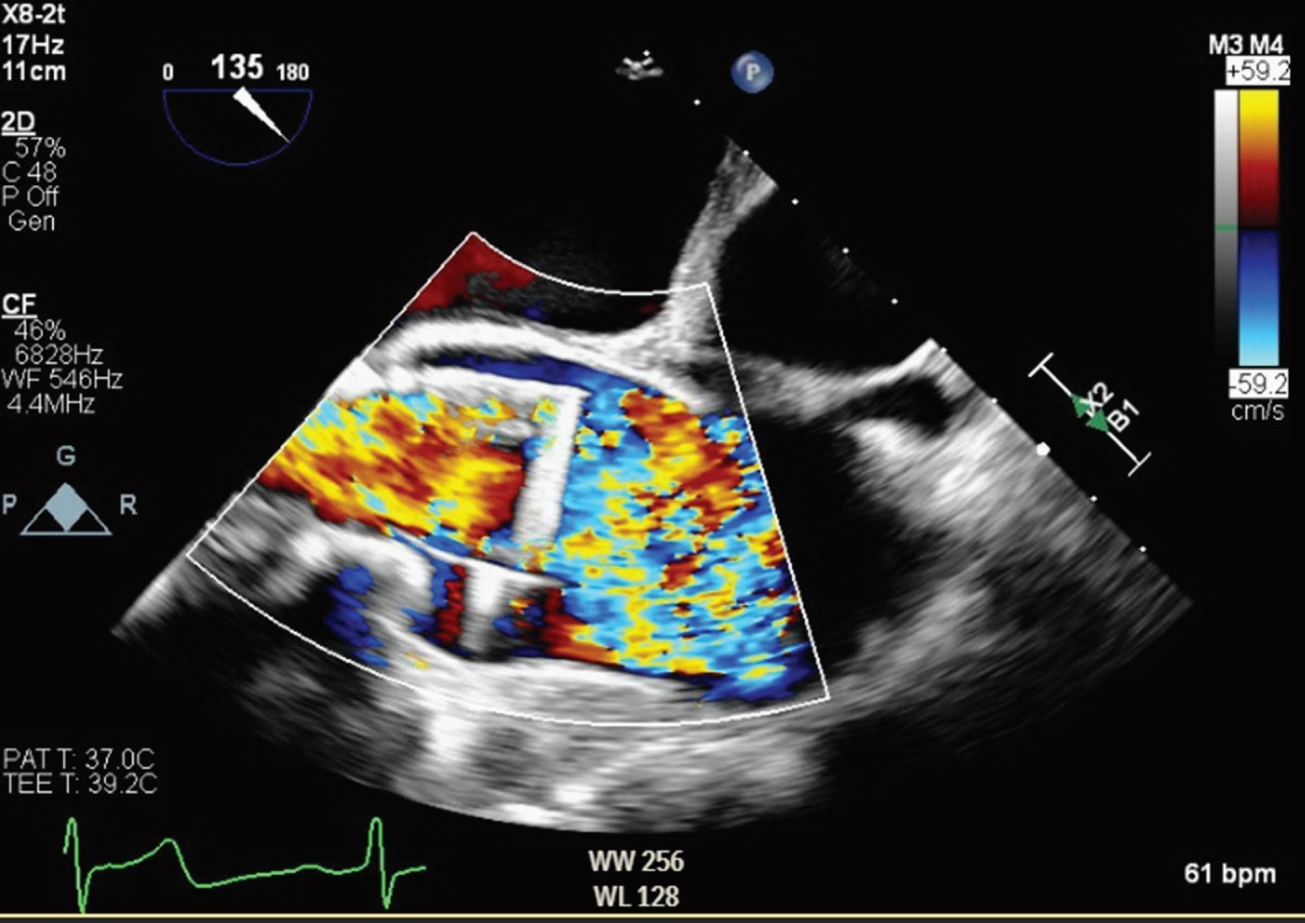
(A)

**Figure:**
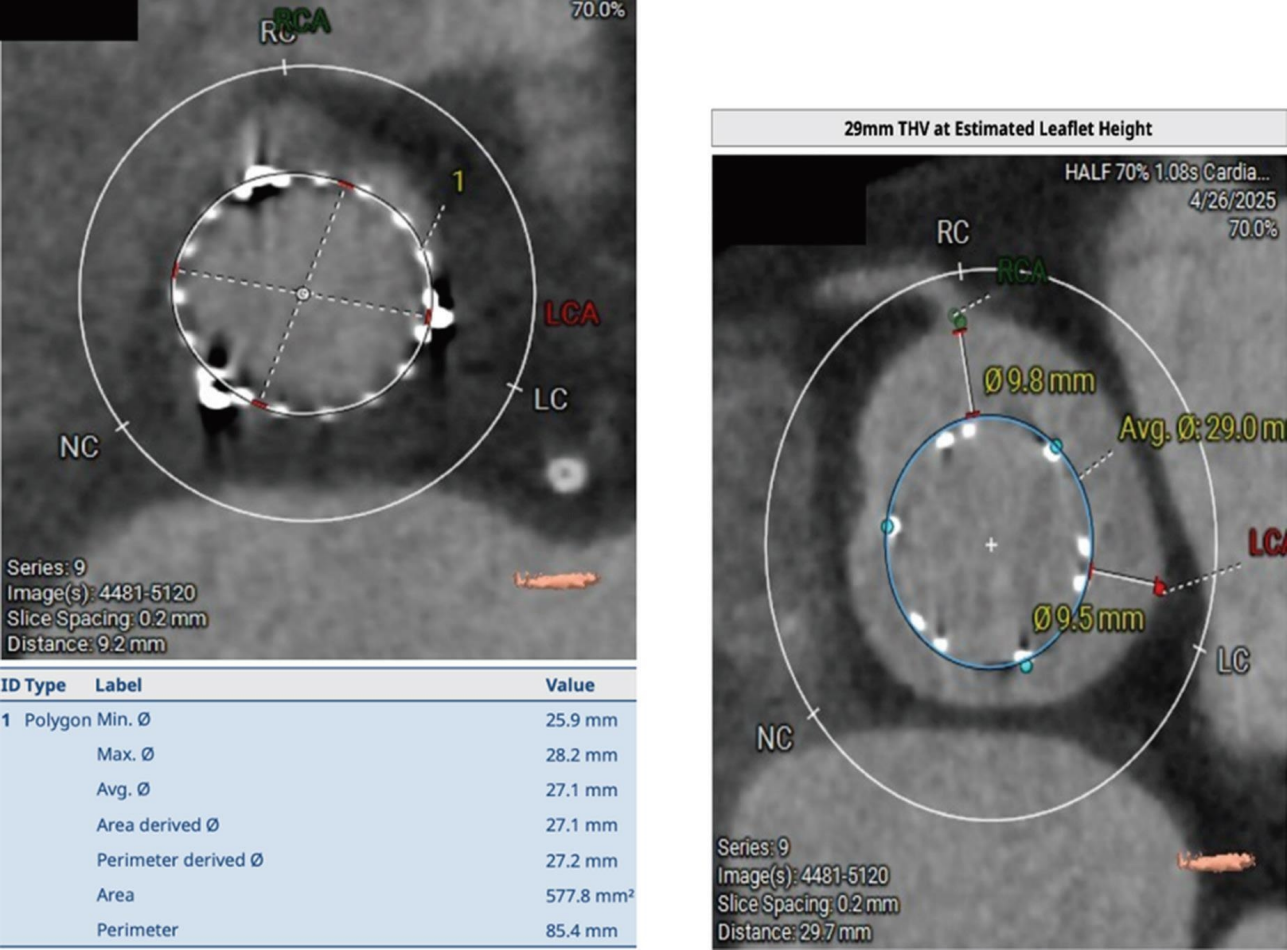
(B)

The patient was taken to the operating room. Following induction and appropriate safety checks, a transvenous pacing system was introduced. The left common femoral artery was accessed primarily and the right secondarily. The Trilogy valve was crossed and aligned to coplanar view in the standard fashion. The LMCA was engaged with a catheter to visualize and line up the S3 valve (Figure 2A) to seat at the level of the neoannulus and to still be below the LMCA. This was deployed (Figure 2B), and aortography demonstrated good position without PVL or significant AI (Figure 2C). The mean gradient was 4 mmHg.

Figure 2(A) Deployment of S3 valve through Trilogy valve with engaged LMCA. (B) Deployed ViV TAVR and demonstrated patency of LMCA. (C) Completion aortography demonstrating LMCA filling and no paravalvular leak or insufficiency. (D) Illustration of the Sapien S3 valve within the Trilogy valve. The S3 valve seats within the trilogy valve at the level of the neoannulus. The LMCA is given contrast for reference. The ultimate location of the Trilogy leaflets was estimated during preoperative planning. Coronary angiography demonstrated there was no obstruction of the coronaries by the Trilogy leaflets. TTE demonstrated no obstruction of outflow with a low gradient across the valve.

**Figure:**
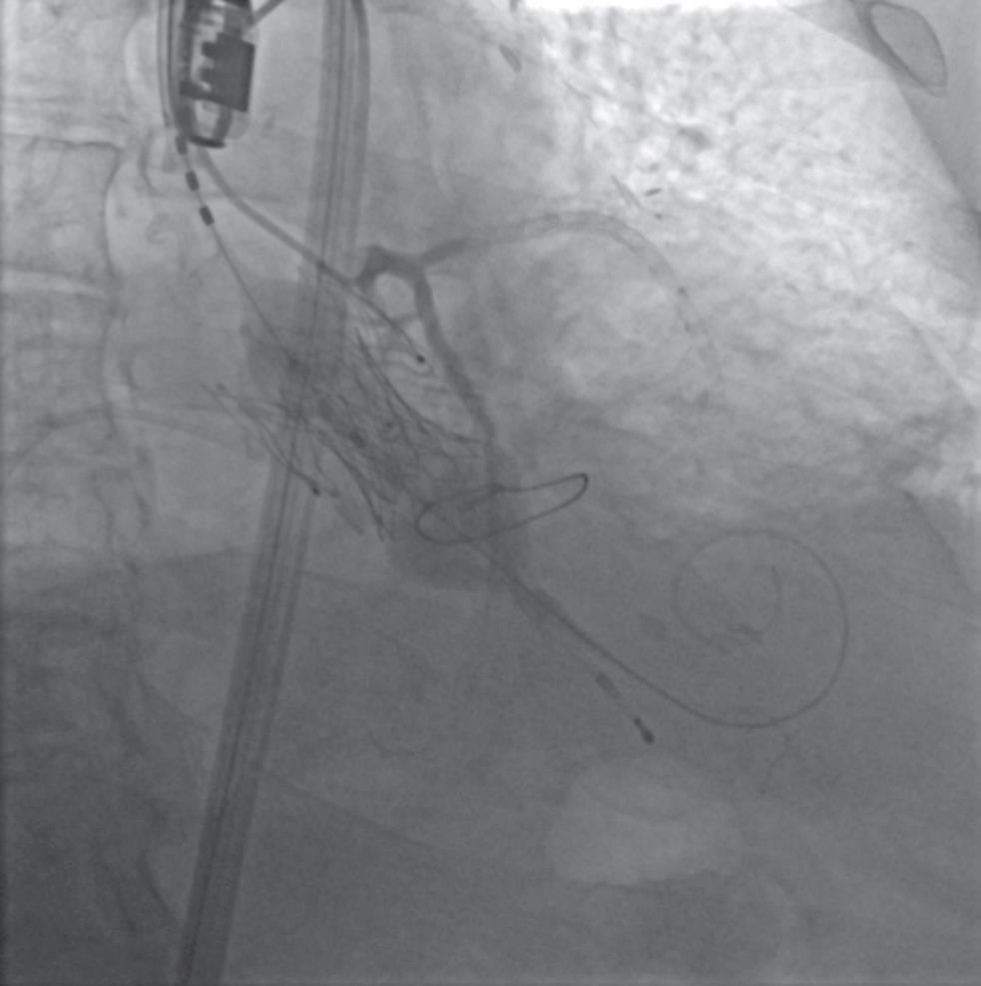
(A)

**Figure:**
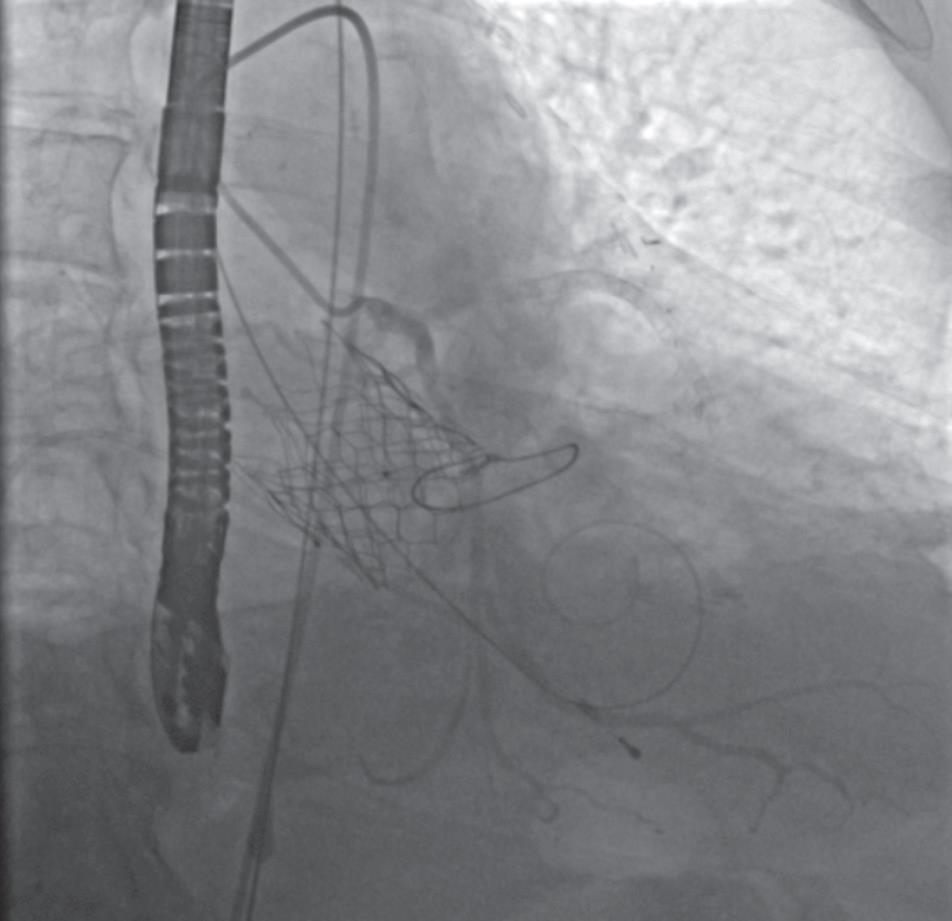
(B)

**Figure:**
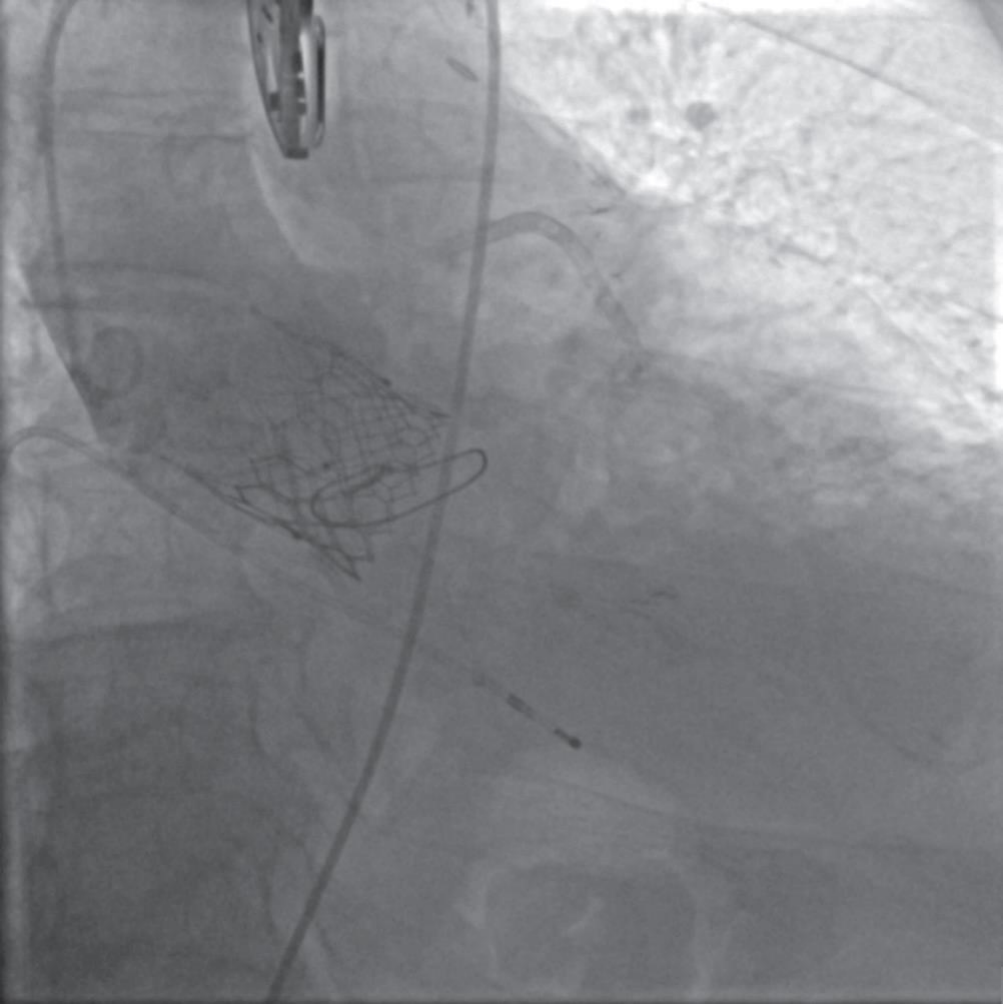
(C)

**Figure:**
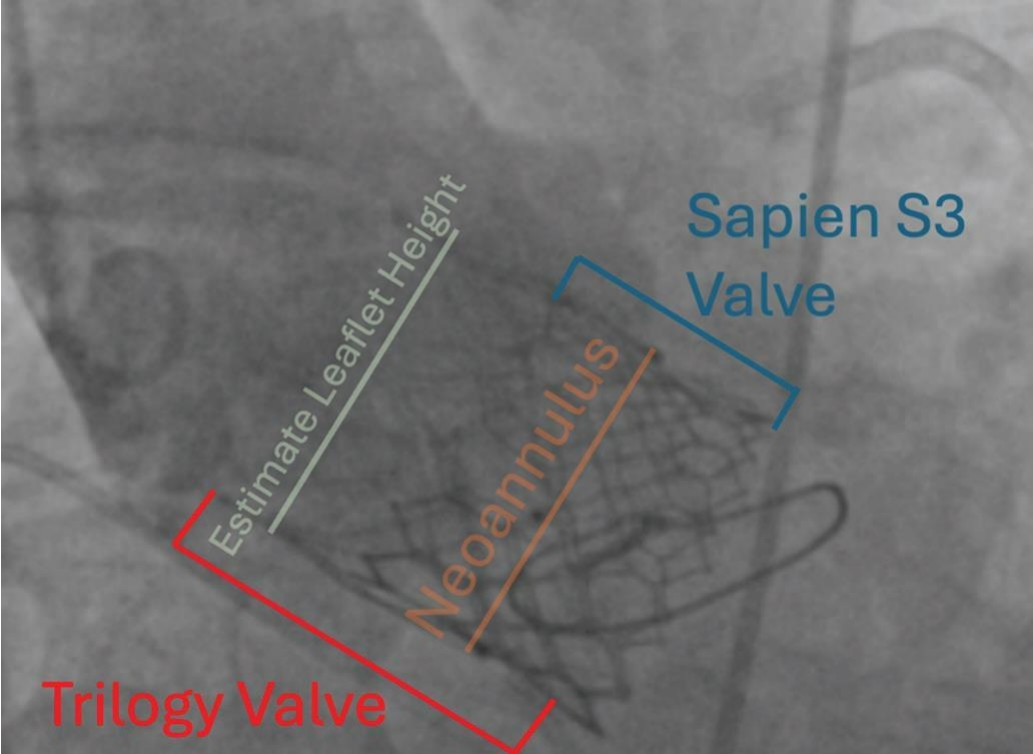
(D)

Postoperatively, the patient developed a new and persistent left bundle branch block, which required dual chamber pacemaker implantation. He otherwise had an uncomplicated course and was discharged to home on postoperative day 6 following device interrogation and management of urinary retention. Follow‐up TTE at 6 months revealed a normal functioning ViV TAVR valve with trivial AI and a mean gradient of 12.9 mmHg.

## 3. Discussion

ViV TAVR has been demonstrated as a safe procedure with the risk of death of 0.6% and risk of 30‐day mortality of 4%. These risk factors are not significantly different than the risk of first time TAVR [4]. Indeed, the number of ViV TAVR cases is steadily increasing each year with 4871 cases performed in 2019 in the United States [5]. These results are exciting; however, until recently the focus of TAVR has been the treatment of AS. With the advent of new valves designed to treat AI that are not dependent on severe calcific disease for their anchoring mechanism, the use of TAVR will likely continue to expand. As such, the frequency of ViV TAVR will also expand.

The mechanism of improvement of AI in this case is multifactorial. The balloon expandable Sapien S3 sits at the level of the native annulus, pushing aside the dysfunctional leaflets and replacing them with the Sapien leaflets. Thus, the transvalvular regurgitation was addressed. The paravalvular leak was improved by ballooning the valve during deployment such that both the Trilogy valve and Sapien were forced to expand fully. One of the noteworthy technical challenges associated with ViV TAVR is managing the risk of coronary obstruction. Although infrequent, obstruction of the left coronary ostium is devastating with a 50% mortality rate at 30 days. This can be caused by displacement of the leaflets of the native or prior TAVR valve, embolism, or mispositioning of the new valve. Risk factors include low‐lying coronary arteries and supra‐annular valves, stentless prosthesis. Careful attention to preoperative imaging and coronary height is critical to prevent this devastating complication [6]. Though the valve was placed within the existing supra‐annular Trilogy valve, LMCA angiography was performed to reassure that the LMCA was not going to be obstructed with the new valve geometry, as the Sapien valve was placed within the Trilogy valve at the level of the neoannulus (Figure 2C).

Another notable complication of ViV TAVR is a pacemaker rate of approximately 6% for TAVR in AS patients [5]. This figure is even higher in TAVR for AI, with a permanent pacemaker implantation (PPI) rate of 24% of patients in the ALIGN‐AR trial for first time TAVR in AI [2]. Though specifics of ViV TAVR for AI are lacking due to paucity of data, the rate of PPI is likely much higher than for ViV TAVR for AS and subsequent failed valve.

In this case report, we report a case of TAVR for AI followed by early valve failure requiring ViV TAVR for recurrent severe AI. We achieved an excellent hemodynamic result following placement of the S3 valve within the Trilogy valve, suggesting transcatheter treatment of AI following TAVR for AI with the Trilogy valve is possible and reasonable in high‐risk surgical candidates.

## Funding

No funding was received for this manuscript.

## Ethics Statement

The authors have nothing to report. Consent was obtained from the patient. Patient informed consent is attached.

## Conflicts of Interest

The authors declare no conflicts of interest.

## Data Availability

Research data are not shared.
